# Deciphering Antibiotic-Targeted Metabolic Pathways in *Acinetobacter baumannii*: Insights from Transcriptomics and Genome-Scale Metabolic Modeling

**DOI:** 10.3390/life14091102

**Published:** 2024-09-02

**Authors:** Fatma Zehra Sarı, Tunahan Çakır

**Affiliations:** 1 Institute of Biotechnology, Gebze Technical University, Gebze 41400, Kocaeli, Türkiye; fzsari@gtu.edu.tr; 2 Department of Bioengineering, Gebze Technical University, Gebze 41400, Kocaeli, Türkiye

**Keywords:** *Acinetobacter baumannii*, antibiotic resistance, metabolic model, reporter metabolites

## Abstract

In the ongoing battle against antibiotic-resistant infections, *Acinetobacter baumannii* has emerged as a critical pathogen in healthcare settings. To understand its response to antibiotic-induced stress, we integrated transcriptomic data from various antibiotics (amikacin sulfate, ciprofloxacin, polymyxin-B, and meropenem) with metabolic modeling techniques. Key metabolic pathways, including arginine and proline metabolism, glycine–serine and threonine metabolism, glyoxylate and dicarboxylate metabolism, and propanoate metabolism, were significantly impacted by all four antibiotics across multiple strains. Specifically, biotin metabolism was consistently down-regulated under polymyxin-B treatment, while fatty acid metabolism was perturbed under amikacin sulfate. Ciprofloxacin induced up-regulation in glycerophospholipid metabolism. Validation with an independent dataset focusing on colistin treatment confirmed alterations in fatty acid degradation, elongation, and arginine metabolism. By harmonizing genetic data with metabolic modeling and a metabolite-centric approach, our findings offer insights into the intricate adaptations of *A. baumannii* under antibiotic pressure, suggesting more effective strategies to combat antibiotic-resistant infections.

## 1. Introduction

*Acinetobacter baumannii* is a Gram-negative, obligate aerobic bacterium and is responsible for nosocomial and community-acquired infections [1]. As an ESKAPE pathogen with increasing multidrug resistance [2], it can lead to severe infections like pneumonia, bacteremia, urinary tract infections, and meningitis, particularly in immunocompromised individuals. The development of resistance to last-resort antibiotics has led the World Health Organization (WHO) to designate it as a “Priority 1: Critical” pathogen [3].

Carbapenems, including meropenem, are β-lactam antibiotics used as a first-line therapy for multidrug-resistant (MDR) *A. baumannii* infections. They disrupt cell wall synthesis by inhibiting penicillin-binding proteins during the final phase of peptidoglycan assembly [4]. However, their prior use has resulted in an increased prevalence of carbapenem-resistant strains [5]. Amikacin sulfate, an aminoglycoside antibiotic, targets *A. baumannii* by binding to the 30S ribosomal subunit, leading to the production of mistranslated proteins [6]. These faulty proteins misassemble in the membrane and are degraded by proteases, aiding in bacterial elimination [7]. Colistin (polymyxin E) and polymyxin-B are the current antibiotics of choice for MDR *A. baumannii* infections when susceptibility testing suggests that carbapenems and aminoglycosides are unlikely to be effective [8]. Polymyxins target the lipid A component of lipopolysaccharides (LPS)/lipooligosaccharides, which are localized in the outer membrane of the bacteria. Disorganization of the membrane causes the leakage of intracellular metabolites [9]. *Acinetobacter* isolates can be susceptible to fluoroquinolone-class antibiotics such as ciprofloxacin. Ciprofloxacin disrupts DNA replication, transcription, and repair by inhibiting DNA gyrase and topoisomerase IV, and it also induces double-strand breaks (DSBs) in DNA [10]. Alternative treatments for *A. baumannii,* including tetracyclines, beta-lactamase inhibitors, and combination therapies, are available [11]. However, understanding the bacterium’s subcellular response to these treatments is crucial, as resistance can develop over time. One powerful approach to this challenge is transcriptome analysis since it enables the identification of genome-wide alterations in the gene expression levels.

Several studies have utilized RNA-Seq datasets to examine the transcriptomic responses of *A. baumannii* to antibiotic stress [12,13,14]. Qin et al. analyzed 12 *A. baumannii* strains with varying antibiotic resistances and found that amikacin up-regulates genes involved in protein folding and lysis, whereas carbapenems down-regulate transcription factor genes [12]. Another study reported that colistin affects the expression of genes related to outer membrane biogenesis in *A. baumannii*, likely due to the disruption of membrane integrity [13]. Li et al. identified common differentially expressed genes (DEGs) in several *A. baumannii* strains under polymyxin treatment, affecting membrane biogenesis and homeostasis, lipoprotein and phospholipid transport, efflux pumps, PNAG biosynthesis, and fatty acid biosynthesis [14]. In addition, sub-inhibitory doses of minocycline primarily altered the expression of the genes related to the chaperonin system, stress response, and transport system [15]. Although these studies have elucidated various cellular responses to antibiotics, they have not specifically addressed metabolic alterations in *A. baumannii*. Given the critical role of bacterial metabolism in antibiotic response [16,17,18], further investigation focusing on metabolic changes is warranted.

Genome-scale metabolic models (GSMMs) provide a comprehensive collection of organism-specific biochemical reactions and predict metabolic changes in bacteria during infection or under antibiotic treatment [19,20,21,22,23]. iAB5075 has the highest genome coverage (1015 genes) and the highest number of reactions (2207 reactions) among the available *A. baumannii* metabolic models [19]. The integration of omics data with GSMMs can enhance prediction quality [20,24], but the literature lacks a systematic and comparative analysis of *A. baumannii*’s metabolic alterations in response to a range of antibiotics using both GSMMs and RNA-Seq data.

In the present study, we analyzed an extensive publicly available RNA-Seq dataset belonging to *A. baumannii* to elucidate the bacterial metabolic pathways and metabolites under antibiotic pressure. This dataset captures the transcriptomic responses of different *A. baumannii* strains to sub-minimum inhibitory concentration (MIC) doses of clinically relevant antibiotics (amikacin sulfate, ciprofloxacin, polymyxin-B, and meropenem). These antibiotics have different mechanisms of action, and we aimed to identify how this is reflected in the alterations of pathways and metabolites. To achieve this, we integrated the data with the most comprehensive genome-scale metabolic model of *A. baumannii* (iAB5075) and compared the responses of five different *A. baumannii* strains to antibiotics, while the control samples received no treatment. Additionally, we identified the reporter metabolites, those experiencing the most significant transcriptional changes across all strains under antibiotic treatment. Considering the close relationship between antibiotic resistance and metabolic alterations [16,17], our findings highlight key metabolic pathways involved in *A. baumannii*’s response to antibiotics.

## 2. Materials and Methods

**Figure 1:**
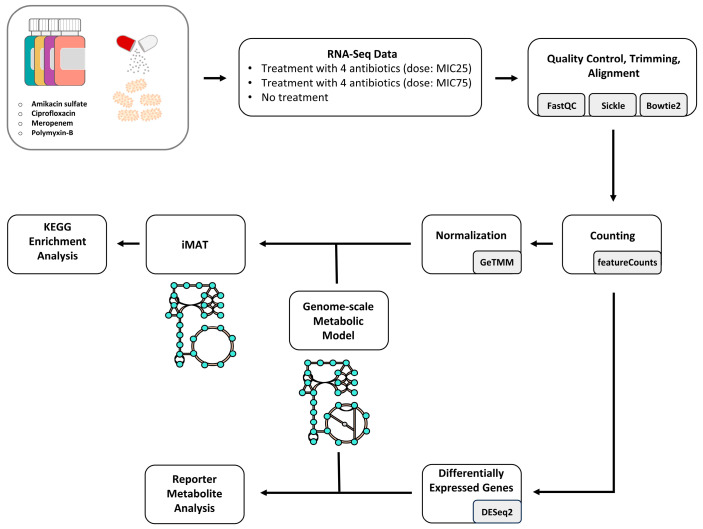
The workflow followed in this study. RNA-Seq data was processed and normalized to map each transcriptomic sample individually in the genome-scale metabolic model. Perturbed reactions in response to antibiotics were used to identify corresponding perturbed KEGG pathways, and DEGs were used to identify reporter metabolites.

The overall methodology followed in this study is shown in Figure 1.

### 2.1. Transcriptomic Data Collection and Analysis

We obtained the raw RNA-Seq data for nine *Acinetobacter baumannii* strains from NCBI’s BioProject public repository (accession number: PRJNA234525). Each strain was represented by 20 samples across 10 distinct experiments, each performed in duplicate, resulting in a total of 200 samples in the dataset. These experiments included treatments with two dosages of four antibiotics (amikacin sulfate, ciprofloxacin, polymyxin-B, or meropenem), a no-treatment control, and an NaCl treatment. The experiments were performed in Mueller–Hinton Broth (MHB), where antibiotic treatments were administered at 25% or 75% of the approximate MIC value of each antibiotic. NaCl-treated samples were excluded from this study.

We aligned the data for each strain (180 samples in total) to the genome assembly of the model strain AB5075 from NCBI (RefSeq assembly accession: GCF_000963815.1) using the Bowtie2 (v2.5.0) [25]. We then proceeded with the five strains that had overall alignment rates exceeding 90%. Table 1 lists the gene expression omnibus (GEO) dataset IDs and strain IDs of these strains. Consequently, we analyzed 90 samples from these five strains, which were treated with four different antibiotics at two different dosages or left untreated.

Transcriptomic datasets were downloaded in FASTQ format through the SRA Explorer platform (https://sra-explorer.info/, accessed on 1 January 2023). Reads were trimmed using Sickle (v1.33) [26] based on the average Phred quality score threshold of 25. After alignment of the trimmed reads onto the reference genome the SAMtools software package (v1.16.1) was used to convert SAM files into BAM files and sort them by chromosomal coordinates [27]. Read counts of gene transcripts were determined using the featureCounts tool (v1.6.0) [28].

### 2.2. Genome-Scale Metabolic Modeling for Transcriptome Mapping

A genome-scale metabolic model (GSMM) of *A. baumannii* AB5075, “iAB5075”, was downloaded in the SBML format [19]. The model includes 2207 reactions controlled by 1015 genes. The model simulations were performed using the COBRA Toolbox (v3.0) [29] and Gurobi optimization software (v 10.0.0) in MATLAB R2022b.

A preliminary assessment of the model revealed ATP leakage by the model. All the uptake reactions in the model were blocked, and the rate of the R_ATPM reaction, the reaction that represents non-growth-associated maintenance (NGAM) energy expenditure, was maximized. A nonzero rate for the reaction pointed to ATP leakage by the model. Next, given that all the uptake reactions were blocked, all the reactions in the model were scanned by single reaction deletion, and the rate of R_ATPM was maximized for each deletion to determine the reaction that stopped ATP leakage when blocked. To speed up the scanning procedure, reactions that were already inactive when all the uptake reactions were blocked were identified using a flux variability analysis [30] and not included in the reaction deletion simulations. As a result, the reaction with ID “R_MGt5”, which transports magnesium from cytosol to the extracellular environment, was detected as the leakage-causing reaction. This reaction was inactivated in all the simulations in this study.

The gene length-corrected trimmed mean of M values (GeTMM) [31] were calculated from the raw read counts of the RNA-Seq datasets using edgeR to normalize for both library depth and gene length [32]. During the normalization procedure, we used a “counts per million” (CPM) threshold to eliminate unexpressed genes (average CPM across samples ≤ 0.5). iMAT was used for transcriptome mapping on the GSMM [33]. The reaction rate of NGAM was set to 8.39 mmol ATP/gDCW/h in iMAT simulations based on the value reported for *Escherichia coli* [34]. *A. baumannii* is an obligate aerobic bacterium; hence, the minimum possible oxygen uptake rate was set to “0.01” to ensure its activity. The minimum growth rate was assigned as “0.1” to prevent its removal from the model in iMAT simulations and to mimic the fact that the reactions involved in macromolecule synthesis will always be active in a cell. Uptake rates of inorganic molecules were constrained to be not higher than 25% of the carbon uptake rate. iMAT uses lower and upper thresholds to find an optimal trade-off between removing low-expression reactions while keeping high-expression reactions. The 25th and 75th quantiles of the average expression levels of genes across all samples in the dataset were used as lower and upper thresholds for creating iMAT-based models. The iMAT function in the COBRA Toolbox was used to run the computational analysis. The metabolic models generated for each sample by iMAT were represented as binary vectors, where zero indicated reactions were removed from the GSMM by iMAT for that sample. Logistic SVD [35] was applied to the binary matrix to produce 3D-PCA plots in R (version 4.3.2), following the removal of the reactions that are active (or inactive) in all the samples from the matrix.

### 2.3. KEGG-Based Pathway Enrichment Analysis

The KEGG pathway annotations for *A. baumannii* were obtained from genome2D (http://genome2d.molgenrug.nl, accessed on 11 March 2023) [36] and eggNOG-mapper [37]. The FASTA file of *A. baumannii* coding sequences was used as input for eggnog-mapper. Annotation data from these two sources were combined, with duplicates and irrelevant metabolic annotations removed. Gene–protein–reaction rules in the GSMM model were then used to link genes to the altered reactions, and these genes were subsequently mapped to associated KEGG pathways. Furthermore, the identified reaction-pathway associations were manually curated for accuracy (Table S1).

Each sample includes two biological replicates in the RNA-Seq datasets. We identified reactions that were either active in both replicates of the treatment group and inactive in both replicates of the control group, or vice versa, to compile a list of perturbed reactions (up-regulated and down-regulated reactions). Enriched KEGG pathways associated with these perturbed reactions were then determined using Fisher’s exact test (*p* ≤ 0.05) (Table S4, Figure S1).

### 2.4. Reporter Metabolite Analysis

Each metabolite in the iAB5075 model was scored using the *p*-values of the genes controlling the reactions that consume or produce that metabolite [38]. The *p*-values were calculated from the raw read counts using the R/Bioconductor package DESeq2 v1.42.1 (DOI: 10.18129/B9.bioc.DESeq2). The *p*-values obtained were first transformed into Z-scores using the inverse of the normal cumulative distribution ($\Theta^{-1}$) function:

(1) $$Z_{ni}=\Theta^{-1}\left(1-p_{i}\right),$$

Then, the Z-score for a metabolite (*Z*_*met*) was determined by combining the Z-scores of the genes controlling the consuming or producing reactions of the metabolite, as described in Equation (2):

(2) $$Z_{met}=\frac{1}{\sqrt{k}}\sum Z_{ni}$$

where *k* is the number of controlling genes for that metabolite. The mean and standard deviation of Z-scores were determined by sampling 10,000 sets of *k* enzymes from the network, thereby normalizing the *Z*_*met* scores (Equation (3)). The corrected Z-scores were then converted back to *p*-values using CDF, identifying metabolites with a minimum of 3 neighbors and *p* value < 0.01 as reporters.

(3) $$Z_{\begin{array}{c} corrected\\ metabolite \end{array}}=\frac{\left(Z_{met}-\mu_{k}\right)}{\sigma_{k}}$$

## 3. Results

### 3.1. Sample-Specific iMAT-Based Metabolic Models from RNA-Seq Data

**Figure 2:**
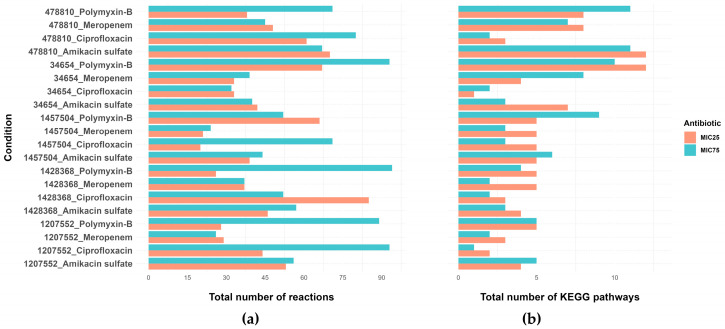
(**a**) Total number of perturbed metabolic reactions and (**b**) enriched KEGG pathways (Fisher’s exact test *p* value ≤ 0.05) for each antibiotic treatment when compared to the control group.

The iAB5075 metabolic model contains 2207 reactions. Using the iMAT algorithm, we generated sample-specific metabolic models with 750 to 896 reactions by identifying and excluding inactive reactions based on RNA-Seq data. We represented iMAT results as binary vectors, where 0 indicated removed reactions and 1 indicated retained reactions for each transcriptomic sample. This approach enabled us to construct a matrix where reactions from all samples were expressed in binary format. We compared binary vectors from each antibiotic treatment group with those from the control group to identify reactions that were removed in both replicates of one group and retained in both replicates of the other. These reactions were classified as perturbed reactions (Table S2). The total number of perturbed reactions was highest for strain 1428368 (*n* = 94) under polymyxin-B-MIC75 treatment, strain 34654 under polymyxin-B-MIC75, and strain 1207552 under ciprofloxacin-MIC75 treatment (*n* = 93) (Figure 2a). Overall, the number of perturbed reactions was between 20 and 94 across the comparisons. The highest number of enriched KEGG pathways was identified for strain 34654 under polymyxin-B-MIC25 and strain 478810 under amikacin sulfate-MIC25 treatment, respectively (*n* = 12, *p* value ≤ 0.05) (Figure 2b).

**Figure 3:**
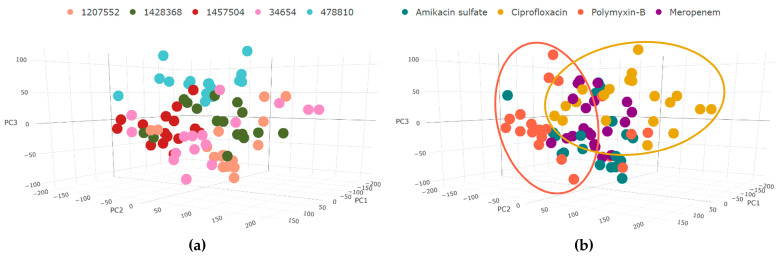
PCA plots of the binarized iMAT models for each sample are shown in (**a**,**b**). (**a**) Labeling with respect to strains, and (**b**) labeling with respect to antibiotic treatment (The proportion of deviance explained is 36% for k = 3).

We used the binary matrix of active/inactive reactions for each transcriptomic sample to create PCA plots, which allowed us to observe similarities and divergences between the samples based on their associated metabolic perturbations. The results of the PCA analyses are presented in Figure 3, separately colored for the strains and the antibiotic types. Strain 478810 was slightly separated from the other strains based on the list of perturbed reactions (Figure 3a), consistent with the findings reported by Li et al. [14]. Their 16S rRNA BLAST and MLST-based analysis classified this strain as a distinct *Acinetobacter* strain. Samples treated with amikacin sulfate and meropenem were clustered closer, while ciprofloxacin and polymyxin-B samples were more scattered and were clearly separated from each other (Figure 3b).

### 3.2. Enriched Metabolic Pathways across Different Antibiotics

We used the genes associated with the perturbed reactions to obtain the enriched KEGG pathways they are involved in (see Methods). The MIC25 and MIC75 results were lumped for each antibiotic before plotting the Venn diagram, and all pathways showing alterations in at least one strain were included (Tables S3 and S4).

**Figure 4:**
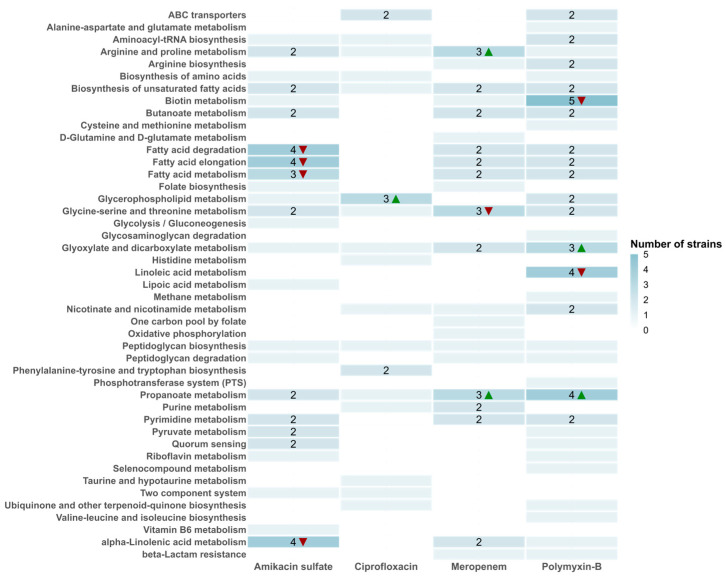
The number of strains with KEGG pathways present includes those that exhibited alterations in response to at least one antibiotic dose. Pathways showing changes in the same direction (Figure S1) in at least three strains are indicated with arrows: red for up-regulated and green for down-regulated pathways.

**Figure 5:**
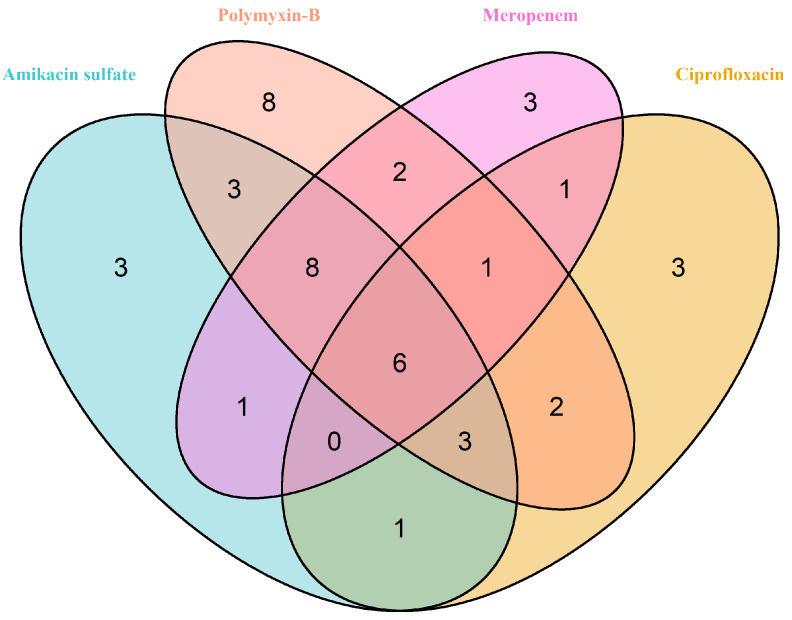
The number of enriched KEGG pathways in *A. baumannii* strains for a given antibiotic.

Antibiotic-target interactions can trigger cellular metabolic shifts as a secondary response to their interaction with their targets. Our results unveiled that across multiple strains of *A. baumannii* exposed to different antibiotics, disruptions were observed in several key metabolic processes. We determined 45 pathway perturbations induced by these antibiotics in *A. baumannii* (Figure 4). Specifically, biotin metabolism (polymyxin-B treatment), fatty acid degradation and elongation (amikacin sulfate treatment), alpha-linolenic acid metabolism (amikacin sulfate treatment), linoleic acid metabolism (polymyxin-B treatment), and propanoate metabolism (polymyxin-B treatment) were significantly enriched in at least four strains (Figure 4). On the other hand, six pathways were commonly affected by four types of antibiotics (Figure 5), with five of these pathways being perturbed in multiple strains (arginine and proline metabolism, glycine–serine and threonine metabolism, biosynthesis of unsaturated fatty acids, propanoate metabolism, and glyoxylate and dicarboxylate metabolism). Additionally, twelve pathways were commonly perturbed by three types of antibiotics, with ten of these pathways affected in multiple strains (Figure 5).

We accessed an additional dataset reporting the transcriptomic response of *A. baumannii* to colistin (GEO Dataset ID: GSE62794) [13] and analyzed it independently to validate our findings. We preprocessed the RNA-Seq samples, derived from 60 min treatment with colistin (0.2 mg), and their corresponding control samples from raw data to GeTMM-normalized counts as detailed in Figure 1. iMAT-based models were constructed and perturbed KEGG pathways were identified. The validation dataset captures perturbations in fatty acid degradation and elongation and arginine metabolisms. Alpha-linolenic acid and quorum sensing were significant pathways in one strain treated by polymyxin-B in our study, and both pathways were significant in the validation dataset. Albeit not significant at the pathway level, there were multiple strains with perturbed reactions from those pathways in the original results. Moreover, reactions from pyrimidine metabolism, glyoxylate, and dicarboxylate metabolism, glycine–serine and threonine metabolism, and propanoate metabolism were identified to be perturbed in the validation dataset. In their original study [13], they only reported two metabolic pathways as perturbed using a differential gene expression approach for colistin treatment: fatty acid degradation and biotin synthesis.

### 3.3. Key Findings from the Reporter Metabolite Analysis

We performed an additional analysis of the transcriptomic dataset using a different metabolism-oriented computational approach: reporter metabolite analysis. Both the iMAT approach and reporter metabolite analysis are based on GSMMs. However, reporter metabolite analysis does not use any mass balance constraints but instead scores each metabolite based on the *p*-values of the genes whose corresponding enzyme consumes or produces that metabolite. The two approaches were complementarily used before [39].

**Figure 6:**
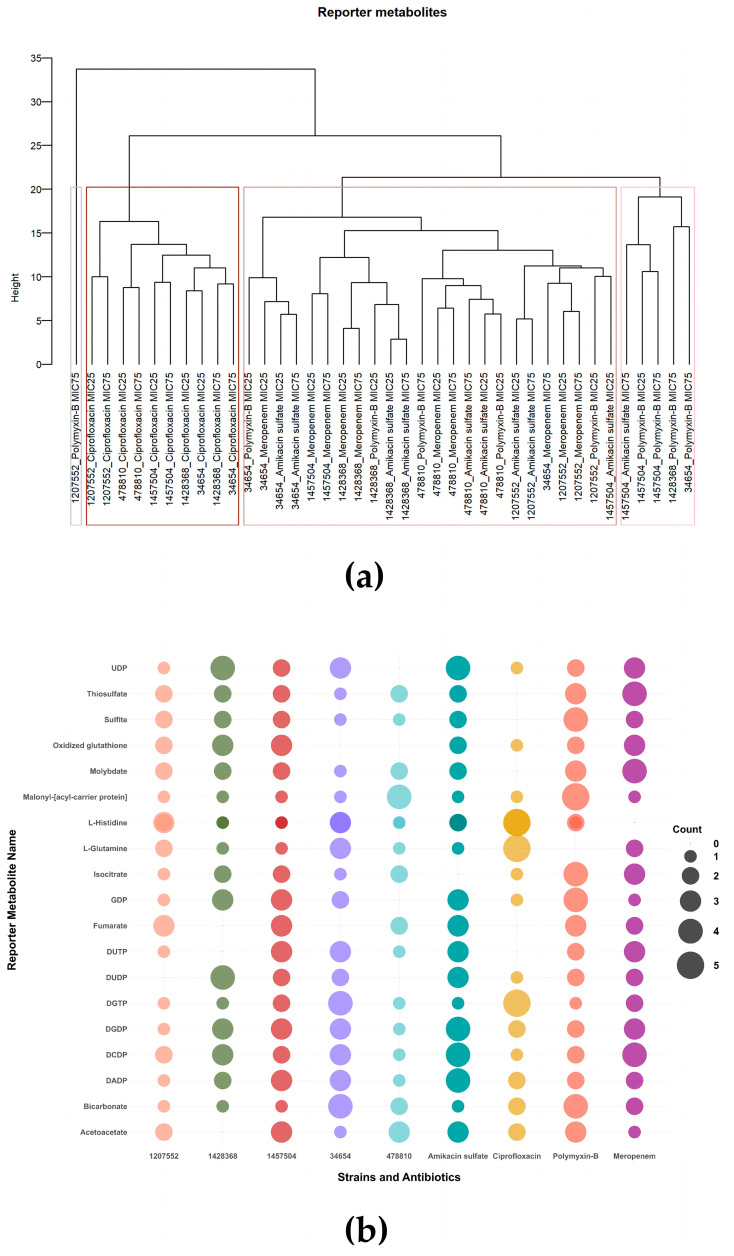
Reporter metabolite analysis results. (**a**) Hierarchical clustering of conditions based on their reporter metabolite profiles, and (**b**) top 20 reporter metabolites, including the number of strains or antibiotics in which they were identified.

We identified reporter metabolites for each condition relative to the control samples. To ensure that the pattern we observed in the PCA graph (Figure 3) was independent of the iMAT approach, we initially performed hierarchical clustering on the identified reporter metabolite profiles (Figure 6a). Consistent with the iMAT results, ciprofloxacin and polymyxin-B are the most distinct antibiotics based also on reporter metabolite analysis. Several conditions involving meropenem or amikacin sulfate treatment, on the other hand, clustered together. Polymyxin-B was distinctly grouped from other antibiotics when iMAT-based up-regulated and down-regulated pathways were identified across different antibiotic dosages, highlighting its unique impact on metabolic profiles (Figure S2). Then, we lumped the reporter metabolites of MIC25 and MIC75 for each antibiotic, similar to the iMAT analysis, and identified metabolites commonly observed across strains or antibiotics (Table S5). The most commonly observed 20 reporter metabolites are given in Figure 6b. Metabolites from purine/pyrimidine metabolisms, the TCA cycle, fatty acid metabolism, and amino acid metabolism dominate the common reporter metabolites.

## 4. Discussion

**Figure 7:**
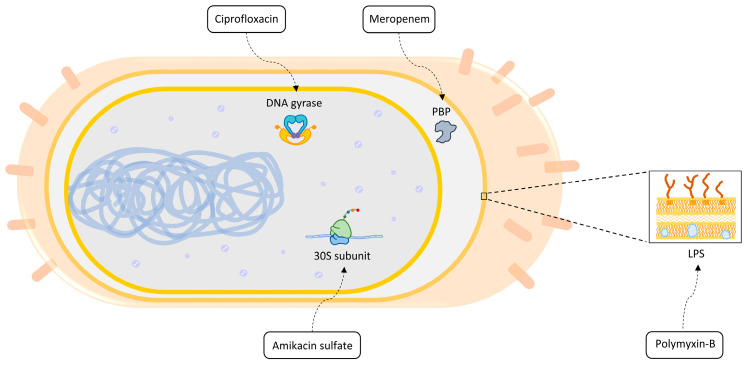
The molecular mechanisms of the antibiotics covered in this study.

In combating *A. baumannii* infections, amikacin sulfate, ciprofloxacin, polymyxin-B, and meropenem are critical antibiotics, each targeting essential bacterial processes (Figure 7). However, the rise of antibiotic resistance necessitates understanding *A. baumannii*’s adaptive responses. This study aimed to elucidate the molecular mechanisms of these responses through a metabolism-oriented investigation. We presented a detailed discussion of perturbed pathways across multiple strains or by various antibiotics to provide insights into the subcellular basis of antibiotic responses.

Biotin acts as a cofactor for several enzymes in lipid metabolism, including acetyl coenzyme A carboxylase and propionyl-CoA carboxylase. The reactions catalyzed by biotin synthase were found to be perturbed in all five strains subjected to polymyxin-B treatment (Figure 4). Notably, a study found that the gene associated with biotin biosynthesis was upregulated over 150-fold in colistin-treated *A. baumannii* [40]. Disruption of this gene increased resistance to colistin, highlighting biotin’s crucial role in responding to membrane perturbation by polymyxin. In line with this finding, a recent study showed that the inhibition of biotin synthesis in *E. coli* restored resistance to colistin [41].

Fatty acid degradation and elongation pathways were significantly perturbed in four strains following amikacin sulfate treatment. These pathways were also affected in two strains when treated with polymyxin-B or meropenem (Figure 4, Table S6). Reporter metabolites such as acetoacetyl-CoA, trans-hex-2-enoyl-CoA, and propanoyl-CoA were consistently identified in multiple strains under amikacin sulfate, polymyxin-B, or meropenem treatment (Table S5). A metabolomics study found significant perturbations in myristic acid and 16-hydroxypalmitic acid levels in *A. baumannii* treated with amikacin [42], with even greater perturbations observed when combined with polymyxin-B. Another study reported that supplementing amikacin treatment with short-chain fatty acids notably reduced biofilm formation and growth in *Mycobacterium avium* [43]. Additionally, pathways for polyunsaturated fatty acids, such as linoleic acid and alpha-linolenic acid, were perturbed in four strains in response to amikacin and polymyxin-B treatments, respectively.

Arginine and proline metabolism, along with glycine–serine and threonine metabolism, were significantly enriched with perturbed reactions across all four antibiotics and multiple strains in our study. Key metabolites of arginine biosynthesis, acetyl-glutamate, and acetyl-glutamate semialdehyde were among the reporter metabolites of amikacin sulfate, polymyxin-B, and meropenem. Phosphoserine, the precursor of serine, was also identified as a reporter metabolite in all antibiotics. Proline metabolism is known to have a major role in the virulence of certain pathogens [44], and arginine was reported to reverse antibiotic tolerance in *E. coli* [16]. Proline has well-known roles in protecting cells from oxidative stress and enhancing protein stability [45]. Both proline and arginine levels were significantly decreased when *A. baumannii* was treated with polymyxin-B combined with rifampicin [46]. A recent study showed that serine reduced the virulence of *A. baumannii* [47]. A proteome-based comparison of multi-drug-resistant and drug-susceptible clinical isolates of *A. baumannii* identified glycine–serine and threonine metabolism as perturbed [48]. Studies have demonstrated that metabolic pathways associated with glycine, serine, and threonine are indeed a critical avenue for adaptation and resistance [49,50].

Glyoxylate and dicarboxylate metabolism and propanoate metabolism were also among the pathways identified to be significantly perturbed in multiple strains by all four antibiotics. Similarly, methyl-isocitrate, a metabolite with a key role in both glyoxylate and propanoate metabolisms, was a reporter metabolite in multiple strains in response to all antibiotics. The role of the glyoxylate cycle in antibiotic resistance was shown before in *E. coli* [51] and in *M. tuberculosis* [52]. The same study on *M. tuberculosis* also reported an association of propionyl-CoA with drug resistance. Mutations in the genes of propanoate metabolism were shown to mediate multidrug tolerance in bacteria in another study [53]. The complementary role of glyoxylate and propanoate pathways on bacterial virulence was also discussed elsewhere [54]. Both pathways were perturbed in *A. baumannii* biofilms, and biofilm formation is a major cause of antimicrobial resistance in *A. baumannii* [55]. When biofilm formation was suppressed in *A. baumannii*, genes of propanoate metabolism were among the major differentially expressed genes [56].

In addition, several reactions associated with the key pathways were identified to be perturbed by multiple antibiotics in multiple strains, although the pathways were not significant in the enrichment analysis. Reactions of pyruvate metabolism are among such reactions. Antibiotics are known to target major energy-consuming processes within the cell [19,57], and pyruvate is a central metabolite for energy by bridging glycolysis and TCA cycle pathways. Alteration in this pathway was previously reported in response to antibiotic treatment in proteomic studies [58,59]. Several metabolites from glycolysis and the TCA cycle were also among the common reporter metabolites, including fumarate, isocitrate, and phosphoenolpyruvate. Reactions of two-component systems and ABC transporters were also among the perturbed reactions in almost all antibiotic applications. Sensing the antibiotic is the initial step in the bacterial defense against external stressors, including antibiotics. Two-component regulatory systems (TCS) in bacteria function as essential sensory pathways that facilitate microbial adaptation to the environment [60]. This system can control the gene expression related to antibiotic resistance and also regulate ABC (ATP binding cassette) transporters [61]. ABC transporters can provide a resistance mechanism to bacteria by exporting the antibiotics outside.

The results presented in this study are predicated upon the analysis of perturbed reactions rather than the conventional examination of differentially expressed genes, the standard methodology in transcriptome data analysis. The inherent advantage of employing genome-scale metabolic modeling lies in its ability to incorporate mass-balance constraints around intracellular metabolites, thereby fine-tuning gene expression data. As intracellular metabolites do not accumulate over time, reactions governing the production of a given metabolite must occur at a rate equal to the rate of reactions responsible for its consumption. The application of iMAT, a genome-scale metabolic modeling tool, capitalizes on these constraints to predict the active/inactive state of reactions from transcriptome data. Therefore, a reaction predicted to be perturbed by iMAT between two compared conditions can hint at post-transcriptional modifications since the genes encoding the enzymes of these reactions will not necessarily be differentially expressed.

One should note that the approach we followed here uses metabolic fluxes (i.e., reaction rates) inherently and identifies perturbed reactions with a conservative approach. In this approach, we only considered reactions that do not carry a flux in one condition (i.e., inactive) while carrying flux in the other condition (i.e., active). There may be reactions that carry flux in both conditions, with a significant difference in the flux values. A flux-prediction-based approach would identify such reactions, leading to a higher number of perturbed reactions and pathways. This may also explain why the reporter metabolite approach identified some commonly regulated metabolites, such as histidine, that were not captured by the iMAT approach. The two approaches use different information to catalog metabolic perturbations. Therefore, they can also be used in a complementary manner.

## 5. Conclusions

Here, we used a genome-scale metabolic model (GSMM)-based framework to investigate metabolic pathways altered in *A. baumannii* in response to four different antibiotics, using an extensive transcriptomic dataset encompassing five different strains. From this dataset, only polymyxin-B had been investigated previouslyby employing the standard approach of identifying differentially expressed genes [14]. Since that study was not metabolism-focused, they only reported perturbations in the generic pathways of amino acid metabolism and fatty acid degradation/biosynthesis. In contrast, several specific metabolic pathways were captured by our analysis for the same antibiotic, indicating the importance of a metabolism-oriented approach.

We have identified key pathways perturbed in multiple strains by the same antibiotic. Understanding these common or antibiotic-specific pathways is crucial for developing new treatments and overcoming antibiotic resistance. Indeed, about a third of current antibiotics target metabolic genes [62], and a strong link exists between bacterial metabolism and antibiotic resistance [17]. Our study provides a detailed catalog of reactions and pathways perturbed by amikacin sulfate, polymyxin-B, ciprofloxacin, and meropenem. Future studies could aim to create broader transcriptomic datasets that include various antibiotics with both similar (other carbapenem or polymyxin types), and distinct mechanisms of action. Additionally, future research might focus on experimentally validating these reactions and pathways as novel targets.

## Figures and Tables

**Table 1:** *A. baumannii* strains used in this study and corresponding GEO (gene expression omnibus) dataset IDs. Each strain had 18 samples from nine different experiments (four antibiotic treatment experiments at MIC25 dosage, four antibiotic treatment experiments at MIC75 dosage, and one experiment without antibiotic treatment). Each experiment was performed in duplicates.

GEO Dataset	*A. baumannii* Strain	Number of Samples Used in This Study
GSE56222	1207552	18
GSE56223	1428368	18
GSE56224	1457504	18
GSE56218	34654	18
GSE56219	478810	18

## Data Availability

The raw data used in this study is available at: https://www.ncbi.nlm.nih.gov/bioproject/PRJNA234525. The processed data presented in this study is available at: https://github.com/SysBioGTU/Abaumannii_antibiotics_FZSari.

## Supplementary Materials

The following supporting information can be downloaded at: https://www.mdpi.com/article/10.3390/life14091102/s1, Table S1: Reactions, associated genes, and pathways of the iAB5075 metabolic model; Table S2: Perturbed reactions in iMAT models; Table S3: Pathways of perturbed reactions; Table S4: *p*-values of the pathway enrichment analysis; Table S5: Reporter metabolites and the number of strains or antibiotics they were found in. Table S6: Pathways perturbed in at least two strains for each antibiotic. Figure S1: Up-regulated and down-regulated KEGG pathways under antibiotic treatments. Figure S2: Hierarchical clustering of up-regulated and down-regulated KEGG pathways across different antibiotic treatment dosages.
