# Supplementary material for: Deciphering Antibiotic-Targeted Metabolic Pathways in Acinetobacter baumannii: Insights from Transcriptomics and Genome-Scale Metabolic Modeling
Source: Life (Basel). 2024 Sep 2;14(9):1102. doi: 10.3390/life14091102 (PMC11433532; doi:10.3390/life14091102)
Supplement: Supplementary file 1 [file life-14-01102-s001.zip › Figure S2.pdf]

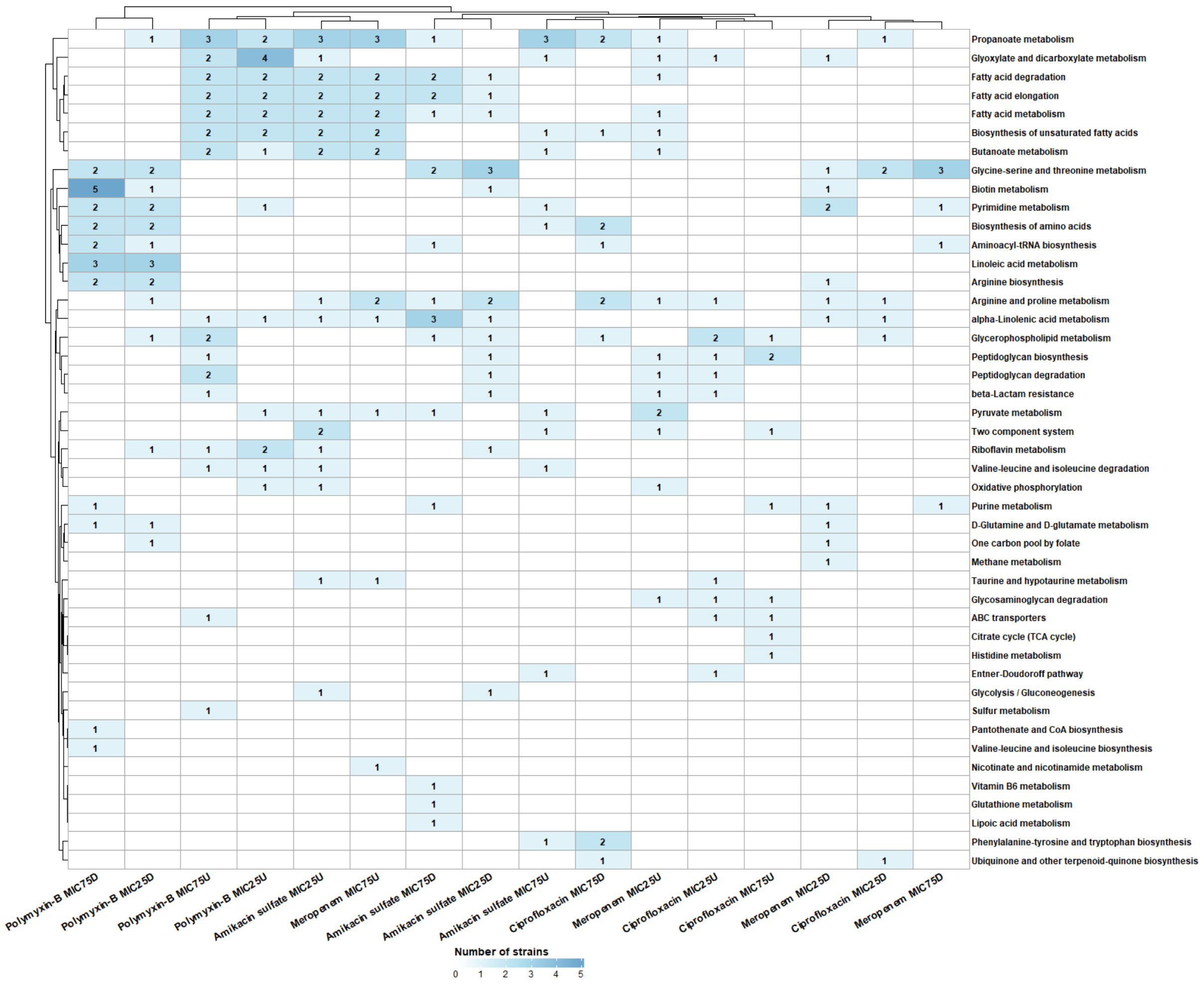

Figure S2. Hierarchical clustering of up-regulated and down-regulated KEGG pathways across different antibiotic treatment dosages. D and U represent down-regulation and up-regulation, respectively.
